# Digital Radiology to Improve the Quality of Care in Countries with Limited Resources: A Feasibility Study from Angola

**DOI:** 10.1371/journal.pone.0073939

**Published:** 2013-09-25

**Authors:** Floriana Zennaro, Joaquim António Oliveira Gomes, Armando Casalino, Magda Lonardi, Meta Starc, Pierpaolo Paoletti, Daniele Gobbo, Chiara Giusto, Tarcisio Not, Marzia Lazzerini

**Affiliations:** 1 Institute for Maternal and Child Health IRCCS Burlo Garofolo, Trieste, Italy; 2 Hospital Divina Providencia, Luanda, Angola; Johns Hopkins Bloomberg School of Public Health, United States of America

## Abstract

**Objective:**

Sub-standard quality in X-ray image acquisition and interpretation is common in low-resource countries, and can ultimately result in higher patient morbidity and mortality. This study aimed at evaluating; 1) feasibility of implementing a digital X-ray device in a second level hospital in Angola; 2) quality of digital X-ray images, when digital radiology was in the hands of local technicians; 3) feasibility of digital teleradiology and its potential impact on case management.

**Methods:**

We developed and tested at the Hospital Divina Providencia (HDP) in Luanda, Angola, a digital X-ray device and a telemedicine network to acquire and print digital X-ray images and send them as DICOM files for remote consultation.

**Results:**

20,564 digital X-ray images were made at HDP from November 2010 to December 2012, with no major technical problems and no need for on-site supervision. Digital radiology largely improved the number of X-ray images of good and very good quality (100% of images with digital radiology, compared to 15% of screen-film images, p<0.0001). Teleradiology using digital images was used in 7.6% of paediatric cases, and provided, in these cases, an important contribution to case management.

**Conclusions:**

The implementation of a digital X-ray device is feasible in low resource settings with significant improvement in quality of X-ray images compared to standard screen film radiology.

## Background

Radiology plays a critical role in the management of many acute and chronic diseases – such as pneumonia, tuberculosis and HIV – in low resources settings. However, in these settings, there is limited access to radiological services of acceptable quality, with massive inequalities in service delivery between the public and private sector [1]–[2].

The standard X-ray technology based on X-ray tube and screen-film is the most commonly used method to acquire X-rays in low resources settings [1]. Nevertheless, in these settings, the quality of screen-film X-ray images is undermined by a variety of factors: a) lack of adequate and equipment and materials (e.g. films, additives) which are costly and difficult to maintain; b) intrinsic nature of the screen-film technology, which requires specialized staff with knowledge and expertise in film developing and processing; c) unavailability of post-processing functions to improve image quality after film development [1]–[2]. Moreover, screen-film images are not designed to exist as files. Screen-film X-ray can't be directly saved, reproduced and circulated for the purposes of patient follow up, nor for external consultation and training. This is particularly relevant in settings characterized by high workload and shortage of human resources [1]–[3]. All these factors may contribute to sub-standard quality in X-ray interpretation, and ultimately result in inadequate case-management, higher patient morbidity and mortality [1]–[3].

Digital technology may be a future solution for many low-resources countries, eliminating the need for film development and processing, being more simple to use, and enabling instant reporting via teleradiology (**Table 1**) [1]–[2]. However, so far digital radiology has been considered too expensive or too sophisticated to use, and its implementation in low resources settings is still very limited, mostly confined to the private sector or to pilot projects [1]–[3].

**Table 1:** Advantages of digital X-ray.

**GENERAL ADVANTAGES OF DIGITAL X-RAY**
Better image quality
Lower cost
Timeliness
Increased dynamic range
No film-developing
No need for water or ventilation for film developing
No hardcopy storage disadvantages
Real-time transferability over long distances
Remote consultation and training
Availability of post-processing functions
**ADDITIONAL ADVANTAGES OF POC 130 OR SYMILAR DIGITAL SYSTEMS**
User-friendly
Compact size
Robustness
High temperature and humidity resilience
Self-diagnosis of internal problems

The Institute for Maternal and Child Health IRCCS Burlo Garofolo has been supporting the Hospital Divina Providencia (HDP) in Angola since 2001, with a focus on paediatric care. The HDP serves a population of about 1 million, in the suburbs of Luanda. Despite recent economic growth following the end of the civil war, Angola still suffers from very high child mortality (mortality rate for children under 5 years is 160/1000), and a widespread lack of essential services [4]–[5]. Only 30% of people in the country have adequate access to healthcare [4]–[5], there is little equipment and too few trained personnel [4]. Total number of doctors in Angola as estimated in 2011 is about 1 doctor for every 10,000 people (1,659 physicians in total) [5]. There is a lack of specialized radiologists, and even fewer doctors are specialized in paediatric radiology.

Before the start of this project in 2010, the HDP provided radiology services though lacking an in-hospital radiologist. It was up to the doctors in charge of patients to interpret the X-rays, based on their individual experience. No system for external consultation, using teleradiology or other methods, was in place.

Since November 2010 external support has been given to HDP from IRCCS Burlo to improve the radiological services. The objectives of this study were to explore: 1) the feasibility of implementing a digital X-ray device in a second-level hospital in Angola; 2) the quality of digital X-ray images, when this device was in the hands of local technicians; 3) the feasibility of a pilot system for remote consultation (teleradiology) based on digital X-rays and its potential impact on case management.

## Methods

### Ethics statement

The Hospital Divina Providencia (HDP) Project Review Board gave the consent to the implementation of the system for acquisition of digital images, and this was implemented from November 2010 as a new standard of care. All X-rays were obtained as part of routine health care, purely for diagnostic purposes based on child complaints. All the children and their mothers gave oral consent to the radiology examinations. The HDP Project Review Board did not required a written consent. The HDP Project Review Board approved the use of oral consent, based on the following considerations: a) the implementation of the digital system does not modify the quantity of X-rays received by the patients, nor does it present any additional health risk for the patient compared to standard radiology; b) radiology examination was performed as part of routine care. Oral consent was documented in the patients' charts. The images were sent from HDP to IRCCS Burlo anonymously, using only patient initials and examination date.

### Equipment

The equipment consisted of: i) a computerized radiography system, that “reads” the X-ray through photosensitive screens and translates them into digital images (i.e. DICOM files, the standard format for digital images); ii) an adequate number of photosensitive phosphor screens.

**Figure 1:**
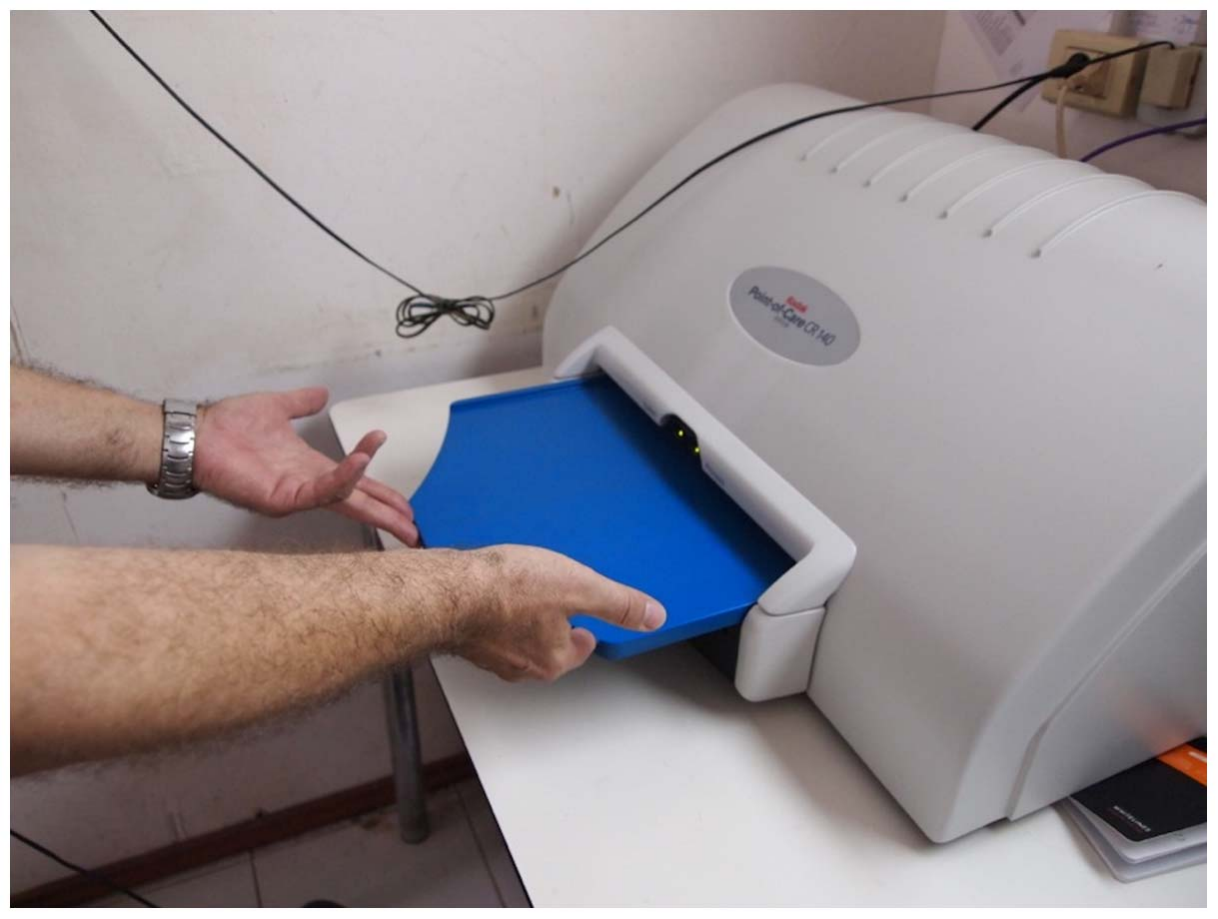
The system for acquisition of digital images (POC 130).

Among the systems for computed radiography available on the market, POC 130 (Carestream®) was chosen for its simplicity, robustness (it can be parachuted into war-zones), low maintenance needs, and cost (26,660 US$) (**Figure 1**). POC 130 is of manageable dimensions (100×50×80 cm), and weight (45 kg). It can work efficiently at a wide range of temperatures (10–40°C) and humidity (90% at 35°C) and can withstand environmental stress such as wind and sand. Compared to standard radiology, it requires neither water nor ventilation to print images. It can self-diagnose and address internal technical problems and it conforms to the International safety and effectiveness technical standards for electrical medical equipment (EN 60601-1, 60825-1, 60601-1-2).

**Figure 2:**
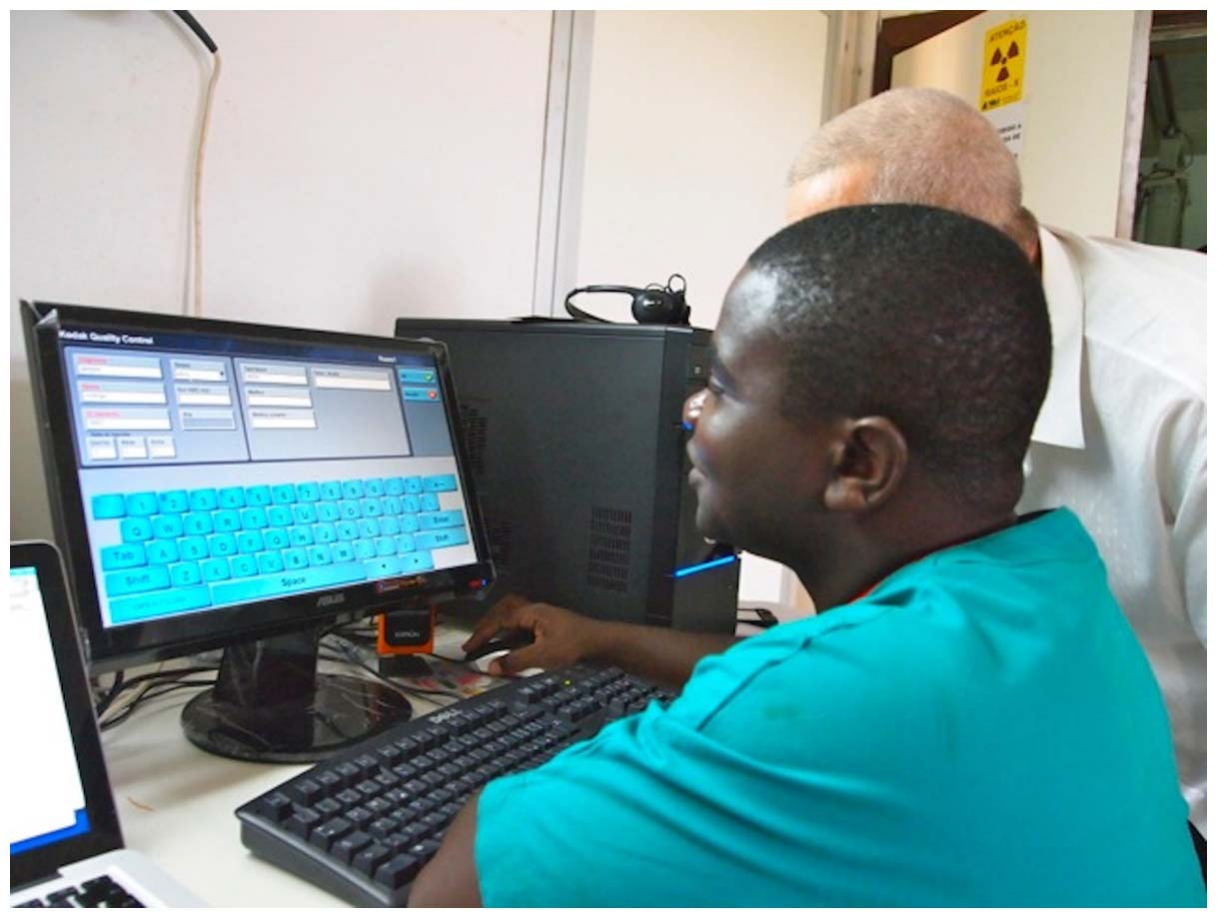
A technician during the training.

In order to operate, the system needs a basic computer (Pentium 4, 2.8 GHz minimum, 1 GB RAM, 80 GB Hard-disk, Microsoft Windows XP operating system) to install the software, which is very simple and user-friendly (**Figure 2**). A 20-inches HP S2031a, with a resolution of 1600×900 dpi was used as monitor.

Image visualization time is shorter than with traditional X-ray (i.e. 60 seconds, compared to 2 minutes), and the maximum throughput reaches 41 images per hour. Images have a high resolution (12 bits/pixel) and can be printed on white paper.

The hospital already had a flat-rate ADSL internet connection. A secured VPN (Virtual Private Network) was set up between the servers of HDP and IRCCS Burlo, to ensure safe data transfer in line with European privacy regulations. The internet line has a speed of 512/256 Kbps and the transmission of a file of about 10 MB usually takes about 2–5 minutes.

The technology was implemented locally by a team composed of an external radiologist (FZ), a radiology technician (PP), and local IT engineer (DG). In Italy an IT technician established the VPN (AC).

### Training

**Figure 3:**
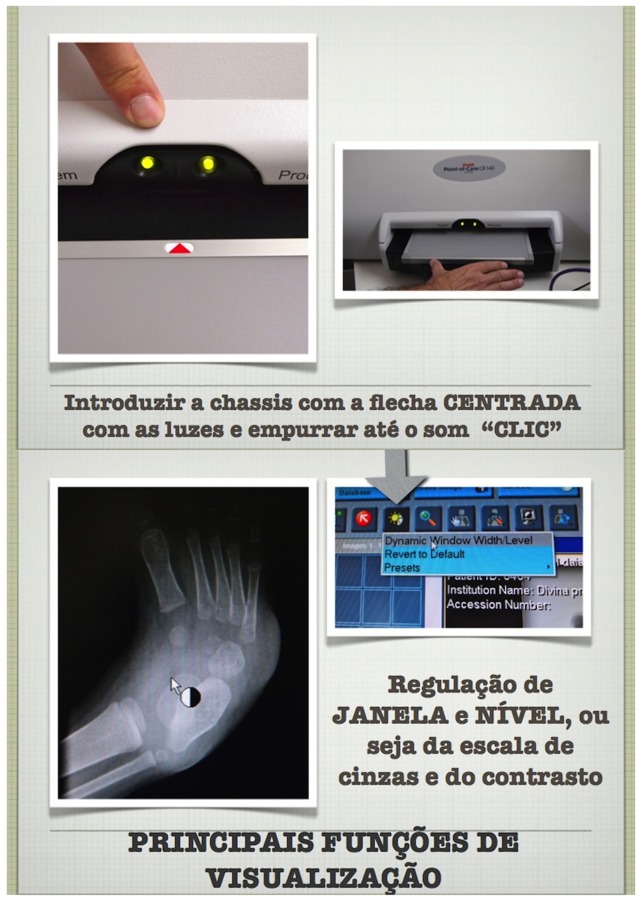
Extract from the training course. Note: This extract from the training course in Portuguese, the national language in Angola, shows how to initiate the system (upper images), and how to choose functions to optimise X-ray visualisation (lower images).

A training course for local staff was organised, with teaching materials in the local language (**Figure 3**). The training included on-site practical sessions.

The total time needed to set up the system and train all the local staff (four radiology technicians) at HDP was ten days.

**Figure 4:**
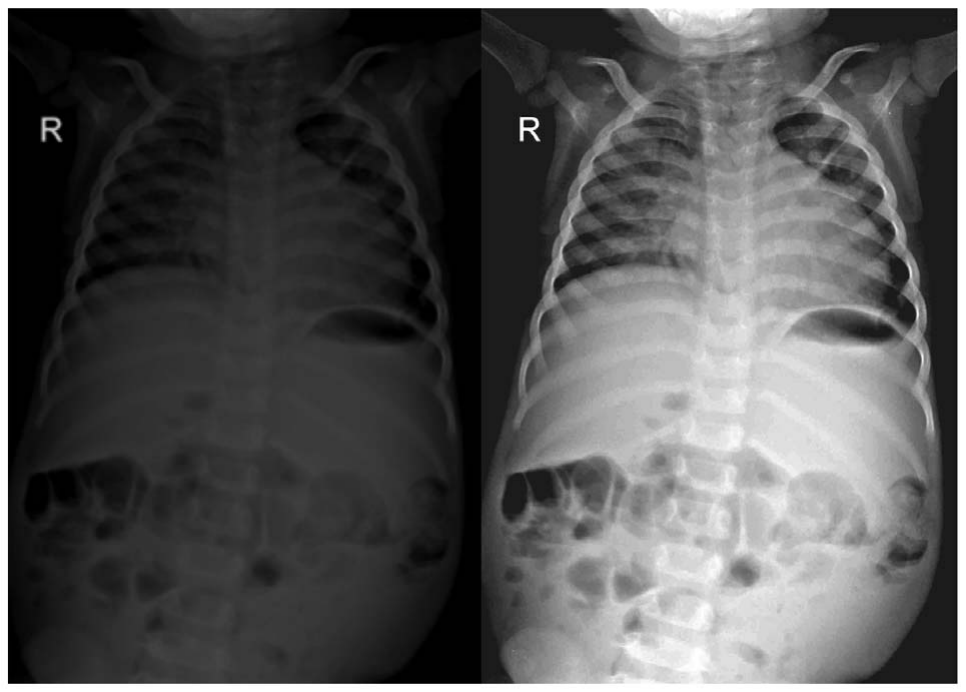
Post processing functions with Digital X- Rays. Note: This is an example of how the quality of digital X-rays could be enhanced by image processing. Depicted on the left is the original X-ray, on the right the same X-ray after digital image manipulation.

**Figure 5:**
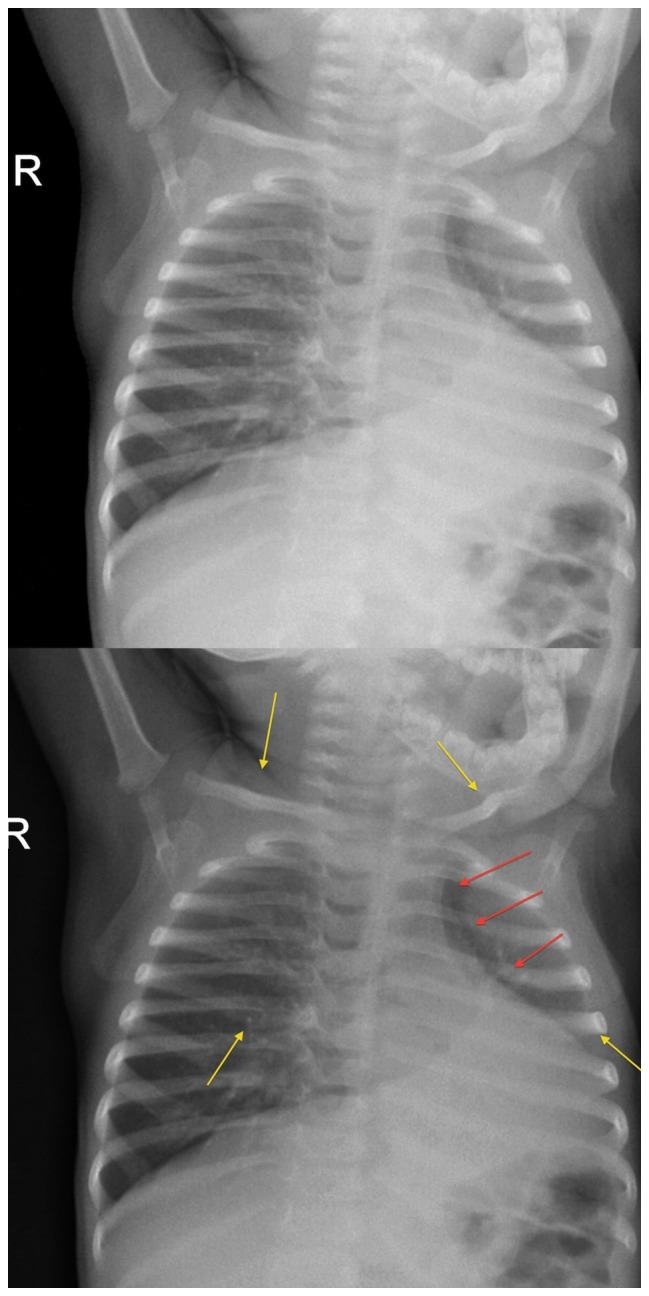
Tutorial for technicians. Note: This and other similar tutorials were produced to teach technicians how to improve image quality through the functions provided by the digital system.

In addition, during the first two months of the study, feedback on the quality of the X-ray images was provided by the external radiologist, and advice was given on how to fully exploit the potential of the digital system, in particular regarding post-processing functions. An example of how the quality of digital X-ray could be enhanced by local technicians by post-processing is provided in **Figure 4**. An example of tutorial for technicians in provided in **Figure 5**.

### Remote consultation

For remote consultation, priority was given to the children cases. All paediatric X-rays judged as “complex” by local paediatricians were sent for external consultation. The staff involved included two out of the five embedded paediatricians working at HDP (ML and MS, routinely in charge of the paediatric ward) and one senior paediatric radiologist at IRCCS Burlo (FZ).

A digital database containing all the images received through teleradiology and child characteristics was prospectively filled in, using FileMaker Bento 4.1.2. A teleradiology report was returned by the radiologist from Burlo to the paediatricians at HDP, free of charge, by e-mail. The local paediatricians, after receiving the external consultation, decided how to manage the cases. Paediatric management was in line with WHO guidelines.

### Analysis

To evaluate local feasibility of digital radiology we looked at: a) any difficulty in implementing the new technology; b) the need for on-site training after the first round of training; c) any reported technical difficulties in maintaining the technology, over two years; d) the volume of work, to establish if there was any drop in volumes of X-ray. Volume of work was calculated by comparing the number of screen film X-rays performed in the three years before the implementation of the digital X-ray system, to the number of digital X-rays performed in the two years following the project implementation.

The quality of X-rays was assessed by two external radiologists working at IRCCS BURLO, not otherwise involved in the project. For obvious ethical reasons it was not acceptable to acquire two X-rays from each child and compare images directly, therefore we judged quality on a random sample of 100 screen-film X-ray images acquired before the implementation of digital radiology, versus a random sample of 100 digital X-rays. X-rays were evaluated on a monitor officially certified for radiologic interpretation, with an image resolution of 2 MB. X-rays were scored by the two radiologists with a score ranging from 0 to 10, which represented four categories of image quality: very good (score 9–10); good (score 7–8); sufficient (score 5–6) and poor (score 0 to 4). The final score was calculated as the mean between the two evaluations from the two assessors. The inter-observers agreement was analysed with kappa statistics (*k*), where a value of *k*>0.6–0.8 represents substantial agreement.

We used means and ranges for numerical variables, frequencies and proportions for categorical variables. Unpaired continuous variables were compared using the two sided t-test. Categorical variables were compared using the two sided chi-square.

We carried out a descriptive analysis of data regarding children at HDP whose digital X-ray images were sent through teleradiology. The contribution of teleradiology to the final diagnosis and case management was defined by three categories: a) “no contribution”, when the interpretation of the X-ray between the two teams was completely or nearly completely consistent; b) “major contribution”, when the two interpretations were completely or largely different; c) “partial contribution”, for intermediate cases.

## Results

### 1. Feasibility

From November 2010 to December 2012 a total of 20,564 X-rays were taken at HDP by local staff, using the digital system, without technical problems nor further need for on-site supervision or retraining. Digital x-ray completely substituted traditional X-ray, both for adults and children. The number of X-rays remained stable through the evaluation period: an average of 10,282±1,397 X-rays/year were made after the implementation of the digital X-ray system, compared to 10,270±2,227 X-rays/year before the digital system was introduced (p = 0.99).

### 2. X-ray quality

**Figure 6:**
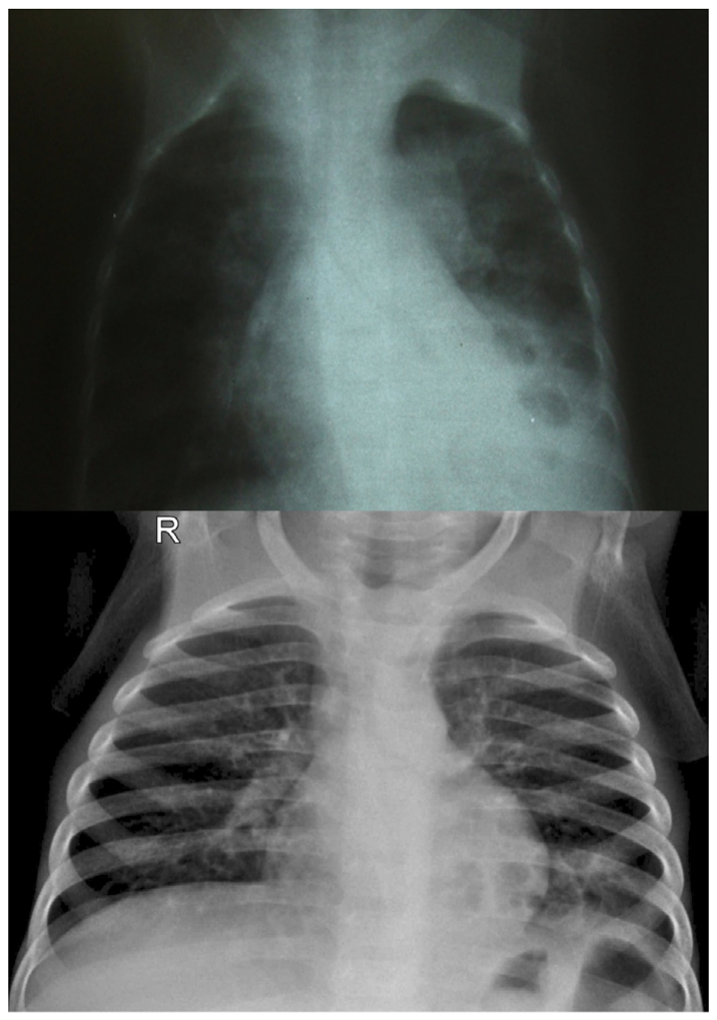
Visual comparison between standard and digital radiology. Note: A typical screen-film X-ray (upper image) in comparison with a digital X-ray (lower image).

There was a significant improvement in quality of X-ray images with the implementation of digital X-rays (**Table 2**). Screen-film X-rays were rated as of poor quality in 42/100 (42%) of cases, of sufficient quality in 43/100 (43%), and of good quality only in 15/100 (15%). Conversely, digital X-rays were rated as of very good quality in 79/100 (79%, p<0.0001), and as of good quality in 21%. Digital X-rays differed from screen-film X-rays in that they were never rated as sufficient or poor quality (p<0.001). K statistics ranged from 0.67 to 0.84. An example of visual comparison between screen-film and digital X-rays is shown in **Figure 6**.

**Table 2:** Quality of X-Rays.

	VERY GOOD	GOOD	SUFFICIENT	POOR
**SCREEN FILM X-RAYS**	0	15	43	2
**DIGITAL X-RAYS**	79	21	0	0

### 3. Teleradiology

Out of 1671 total paediatric X-rays assessed by the two paediatricians involved in the piloting of teleradiology, 127 (7.6%) cases were sent for external consultation. Of these, the vast majority (93.7%) were chest X-rays. Time needed for external consultation never exceeded 24 hours, and ranged from a few minutes to a maximum of 24 hours. A major contribution to clinical management came from external consultation via teleradiology in 49 (38.6%) children, while a partial contribution was given in 58 (45.7%) children (**Table 3**).

**Table 3:** Characteristics of children studied with teleradiology.

VARIABLE OF INTEREST			N = 127
**Age**	Years	Mean (± SD)	2.9 (3.2)
**Sex**	Female	N (%)	58 (45.6)
	Male	N(%)	69 (54.3)
**X-Ray type**	Chest X-ray	N (%)	119 (93.7)
	Skeletal	N (%)	8 (6.3)
**Contribution of teleradiology to case management**	No contribution	N (%)	20 (15.7)
	Minor contribution	N (%)	58 (45.7)
	Major contribution	N (%)	49 (38.6)

## Discussion

Our study has some limitations. It is mainly a feasibility study, and it did not aim at evaluating either the impact of digital radiology on health outcomes, or its cost-effectiveness. The project's primary objective was to document the feasibility of implementing a device for digital X-ray in a low resources setting, and to evaluate its impact on the quality of the X-ray images. After having tested the feasibility of implementing a new technology, the evaluation of its impact on health outcomes, and, if relevant, its cost-effectiveness, may follow as a subsequent evaluation.

This experience proved that the implementation of a low-cost digital X-ray device similar to what we have used in this project is feasible in low resource settings, and that such a device in the hand of local technicians significantly improved the quality of X-ray images.

This project presents three main innovative features. First, the project aimed at implementing a “real” digital system, which made digital images directly available for routine day care. The majority of other experiences [6]–[12], even the very recent ones, used screen-film images converted into digital files by using digital cameras, scanners, or specialized digitizer. Such systems are feasible, but require extra work for converting the X-ray films into a digital file [13]. Moreover, any “digital conversion” system has limited space to improve the image quality if the primary X-ray is not of sufficiently good quality [7]–[9].

This project showed that, following the implementation of digital radiology, only images of good or “very good” quality were produced by local technicians. Technicians realised that the quality of their work had increased, which made them proud and brought them closer to the new technology. The new technology was simple, easy to manage and quick. There are several supposed advantages of digital radiology (**Table 1**), however we believe that these characteristics (i.e. capacity to increase the quality of work, simplicity, user-friendliness, timeliness) are probably the key to a successful uptake of any new technology.

Secondly, modern low-cost devices for digital radiology haven't been tested in Sub-Saharan Africa [1]–[14]. Thanks to modern technology, devices for digital radiology are now much more affordable that what is commonly believed [1]–[14]. In our project the cost of purchasing the equipment was 26,660 US$. This initial investment was paid back in two years by eliminating the need for radiographic films and reagents, (which are extremely costly). In the middle and long term the implementation of digital radiology may even produce a net cost saving compared to maintenance cost of traditional screen-film radiology (films, developer, fixer), and this is not a minor consideration in low resources settings. A formal economic evaluation would be needed to fully assess the economic implications of introducing such a device in a resource-limited setting.

Lastly, to our knowledge there are no previous published studies reporting on teleradiology using directly acquired digital images (DICOM files), in Sub-Saharan African countries. We performed a search in Pubmed, but we were only able to identify studies that used traditional screen film radiology converted into digital files [1], [7], [9], [10], [11], [14], [15]. Digital radiology and tele-radiology is still at the very early stage in Sub-Saharan Africa [1], [15]. Compared to traditional screen film radiology, digital radiology requires no additional time and efforts to convert the images in digital files, and in this sense it largely facilitates image exchange and remote consultation.

In our project teleradiology was used in selected children (7.6%), although in these cases it was an important instrument to provide external support on diagnosis and case management. The percentage of cases sent for external consultation in our project was comparable to what reported by other telemedicine projects in Africa [16], but higher than in similar experience in India [17]. In each local context, the number of cases sent for external consultation may be affected by several factors, including case mix, local capacities of the staff involved, the efficiency of the system, and the fact itself that a new “service” is introduced.

As documented by other wider experiences [16]–[17] and by a recent systematic review [18], ambitious telemedicine projects are not easy to sustain in the long term, as they may be challenged by different factors: (1) legal factors; (2) sustainability factors; (3) cultural factors; and (4) contextual factors. Based both on the background difficulties in implementing ambitious telemedicine service and on the results of this pilot study, we think that, in the setting were this study was performed, the primary role of teleradiology should be facilitating training and capacity building, rather than providing an external opinion on a large number of cases. In order to strengthen knowledge in X-ray interpretation, existing manuals, such as the *WHO Manual of Diagnostic Imaging, Paediatric Examination* [19], and the *WHO manual of diagnostic imaging, radiographic anatomy and interpretation of the chest and the pulmonary system* [20], may be used in the future as reference training materials. In cities with a reasonably good internet connection, such as Luanda, teleradiology could also be used to connect district hospitals to central hospitals, in order to: a) enable local experienced radiologist to perform remote consultation; b) facilitate case-referral and continuity of care; c) facilitate local training and in-country networking.

## Conclusions

The implementation of a low-cost digital X-ray device is feasible in low resource settings, and this can significantly improve the quality of local X-ray images.

## References

[1] Andronikou S, McHugh K, Abdurahman N, Khoury B, Mngomezulu V, et al (2011) Paediatric radiology seen from Africa. Part I: providing diagnostic imaging to a young population. Pediatr Radiol 41: 811–25. 21656276 10.1007/s00247-011-2081-8

[2] Andronikou S, Mngomezulu V (2011) Paediatric radiology seen from Africa. Part II: recognising research advantages in a developing country. Pediatr Radiol 41: 826–31. 21553039 10.1007/s00247-011-2080-9

[3] Jarvis L, Stanberry B (2005) Teleradiology: threat or opportunity? Clin Radiol 60: 840–5. 16039919 10.1016/j.crad.2005.04.001

[4] Angola Country Profile Unicef (2012) Available: http://www.unicef.org/infobycountry/angola_statistics.html. Accessed 2012 Nov 8.

[5] Economic Policy Research Institute. Country Profile: Angola. Available: http://epri.org.za/wp-content/uploads/2011/03/2-Angola.pdf. Accessed 2012 Nov 8.

[6] Al-Shorbaji N, Geissbuhler A (2012) Establishing an evidence base for e-health: the proof is in the pudding. Bull World Health Organ 90: 322–322A. 22589560 10.2471/BLT.12.106146 PMC3341705

[7] Coulborn RM, Panunzi I, Spijker S, Brant WE, Triviño Duran L, et al (2012) Feasibility of using teleradiology to improve tuberculosis screening and case management in a district hospital in Malawi. Bull World Health Organ 90: 705–711. 22984316 10.2471/BLT.11.099473 PMC3442390

[8] Javadi M, Subhannachart P, Levine S, Vijitsanguan C, Tungsagunwattana S, et al (2006) Diagnosing pneumonia in rural Thailand: Digital cameras versus film digitizers for chest radiograph teleradiology. Int J Infect Dis 10: 129–35. 16243559 10.1016/j.ijid.2005.01.007 PMC7110458

[9] Meade K, Lam DM (2007) A deployable telemedicine capability in support of humanitarian operations. Telemed J E Health 13(3): 331–40. 17603836 10.1089/tmj.2006.0040

[10] Corr P, Couper I, Beningfield SJ, Mars M (2000) A simple telemedicine system using a digital camera. J Telemed Telecare 6(4): 233–6. 11027126 10.1258/1357633001935293

[11] Corr P (1998) Teleradiology in KwaZulu-Natal. A pilot project. S Afr Med J 88: 48–9. PMID: 9539937. 9539937

[12] Szot A, Jacobson FL, Munn S, Jazayeri D, Nardell E, et al (2004) Diagnostic accuracy of chest X-rays acquired using a digital camera for low-cost teleradiology. Int J Med Inform 73: 65–73. 15036080 10.1016/j.ijmedinf.2003.10.002

[13] Salazar AJ, Camacho JC, Aguirre DA (2012) Comparison between different cost devices for digital capture of X-ray films: an image characteristics detection approach. J Digit Imaging 25: 91–100. 21614654 10.1007/s10278-011-9391-0 PMC3264722

[14] Shiferaw F, Zolfo M (2012) The role of information communication technology (ICT) towards universal health coverage: the first steps of a telemedicine project in Ethiopia. Glob Health Action 5: 1–8. 10.3402/gha.v5i0.15638 PMC3318899 22479235

[15] Nyathi T, Chirwa T, van der Merwe D (2010) A survey of digital radiography practice in four South African teaching hospitals: an illuminative study. Biomed Imaging Interv J 6: e5. Epub 2010 Jan 1. 10.2349/biij.6.1.e5 PMC3097796 21611065

[16] Zachariah R, Bienvenue B, Ayada L, Manzi M, Maalim A, et al (2012) Practicing medicine without borders: tele-consultations and tele-mentoring for improving paediatric care in a conflict setting in Somalia? Trop Med Int Health 17: 1156–62. 22845678 10.1111/j.1365-3156.2012.03047.x

[17] Singh M, Das RR (2010) Four years of experience of telemedicine for paediatric care in three Punjab hospitals, North India: achievements and lessons. Postgrad Med J 86: 688–91. 20870650 10.1136/pgmj.2009.082735

[18] Saliba V, Legido-Quigley H, Hallik R, Aaviksoo A, Car J, et al (2012) Telemedicine across borders: a systematic review of factors that hinder or support implementation. Int J Med Inform 81: 793–809. 22975018 10.1016/j.ijmedinf.2012.08.003

[19] WHO (2010) The WHO Manual of Diagnostic Imaging: Paediatric Examinations.

[20] Ellis SM, Flower C (2006) The WHO manual of diagnostic imaging: radiographic anatomy and interpretation of the chest and the pulmonary system. Edited by Ostensen H, Pettersson H. WHO. Available: http://whqlibdoc.who.int/publications/2006/9241546778_eng.pdf. Accessed 2013 Jan 30.

