# Correction: Digital Radiology to Improve the Quality of Care in Countries with Limited Resources: A Feasibility Study from Angola

**DOI:** 10.1371/annotation/2bc334cb-4185-42b4-9096-2a7a9adbc7b8

**Published:** 2013-10-04

**Authors:** Floriana Zennaro, Joaquim António Oliveira Gomes, Armando Casalino, Magda Lonardi, Meta Starc, Pierpaolo Paoletti, Daniele Gobbo, Chiara Giusto, Tarcisio Not, Marzia Lazzerini

There were errors in the Author Contributions section. A correct version of the section is available below.

Conceived and designed the experiments: FZ M.Lazzerini. Performed the experiments: FZ JAOG AC M.Lonardi MS PP DG CG. Analyzed the data: M.Lazzerini FZ. Contributed reagents/materials/analysis tools: FZ M.Lazzerini TN. Wrote the paper: M.Lazzerini, FZ. 

